# The elderly and the right to an active aging: the strategy of social cohousing to counteract relational poverty

**DOI:** 10.3389/fsoc.2024.1447614

**Published:** 2024-12-30

**Authors:** Letizia Carrera

**Affiliations:** Department of Research and Humanities Innovation (DIRIUM), University of Bari Aldo Moro, Bari, Italy

**Keywords:** aging process, relational poverty, urban policy, senior cohousing, active aging

## Abstract

The older citizens represent a portion of the population that is not only already high but is also expected to increase according to trend analyses from major national and international research reports. The pandemic experience has shown how they feel the scarcity of relationships and the loneliness of their homes as factors that significantly impact the quality of their daily lives. This challenging historical moment has provided an opportunity to implement a series of projects specifically dedicated to those over 65, aiming to ensure a full range of possibilities for them, starting from highly “enabling” processes. In this perspective, the theme of social senior housing and its various manifestations represents a key strategy to counteract relational poverty, toward intervening in the level of social sustainability through multi-actor network pathways that bring together institutions, private entities, third sector, and research centers. The article aims to analyze the potential that the senior cohousing model can offer, as well as the difficulties this type of shared living arrangement faces—moreover when designed specifically for older individuals and supported by public administrations—, and which still makes it an almost experimental experience in Italy.

## 1 Introduction

The progressive aging of the population is a global phenomenon, but it poses a particularly acute challenge for Europe. This is because in these countries, the percentages are more significant due to the combination of declining birth rates and increasing life expectancy. These trends are clearly reflected in data from various national and international agencies and show no signs of reversal (WHO, UN, Eurostat, Istat). According to data presented by the World Health Organization (2022), the global population aged over 60 will reach ~2,000,000,000 individuals by 2050. In Italy, over just the last 5 years, there has been a 1% increase in the population over 65. The average age of the population has risen from 45.7 years at the beginning of 2020 to 46.5 years at the beginning of 2023. As of January 1, 2023, individuals over 65 number 14,177,000, constituting 24.1% (almost a quarter) of the total population,^1^ and historical data confirm a growing trend.

Moreover, this increasing number of elderly individuals is concentrating their residential choices in cities,^2^ emphasizing the need to correlate the needs expressed by the elderly with urban public policies (Walker and Foster, 2013; Walker and Maltby, 2012). The combination of aging and urbanization processes is already having a significant impact on cities. Elderly individuals look to institutions and administrations as privileged interlocutors tasked with implementing effective, specific, and widespread interventions to ensure high levels of quality in everyday life. These complex dynamics contribute to the growing role of cities as key actors in implementing broad and integrated welfare policies (Ciaffi et al., 2020). While cities did not appear as subjects of international law, which addressed only states as of about 20 years ago, they now emerge as fundamental interlocutors to whom the 2030 UN Agenda attributes a high responsibility in pursuing real equality of opportunities and in formulating and implementing inclusive strategies for recognized elderly subjects, among other socially “vulnerable” categories, considered to be more at risk (Fini et al., 2023; Settersen and Angel, 2011; Bonoli, 2004; Esping-Andersen, 2000; Eping-Andersen, 2012; Emerijck, 2002; Pavolini, 2001, 2004; Zajczyk, 2018; Lalive d'Epinay et al., 2000).

## 2 The aging process as a social challenge

“If chronological age is mathematical, the concept of aging is fluid,” writes Saggio (2022, p. 7), and above all, it is profoundly changing. Beyond the demographic aspect, a qualitative shift has progressively taken shape concerning representations and self-representations of the elderly condition. It is no longer seen as a residual phase compared to the active life but rather as a potentially rich phase of opportunities and occasions. It is an age to invent and experience (Carrera, 2020, 2024), a “third time” to explore possibilities previously unheard of and unthinkable (Ravera, 2023). This goes well-beyond the idea of aging as an “illness in itself” and instead recognizes the hallmarks of aging and contextual elements as factors capable of improving the duration of a healthy life (Rowe and Kahn, 2015; Stuck et al., 1999; Baltes and Mayer, 1999; Alwin and Hofer, 2011; Fries, 2002; Guralnik and Ferrucci, 2003; Ferrucci et al., 2003; Bowling and Stafford, 2007; Silverstein and Giarrusso, 2010; Saggio, 2022). Within the framework of this new social model, conditions are being defined for elderly individuals to imagine something profoundly different from the past, even explicitly claiming a right to a quality of life that goes beyond health. The change in social representations and self-representations among elderly individuals themselves has led to a corresponding shift in expectations, orienting the anticipation of a “new normality.” While loneliness was once considered an inherent part of the condition of being elderly, it has gradually come to be perceived as a problem. Both institutions, which have coined the term “relational poverty” and made its mitigation a focal objective at various territorial levels (World Health Organization, 2015, 2022), and individual subjects, who consider it an important factor for ensuring a higher degree of quality of life and daily wellbeing, now recognize this issue.

This new, broader, more complex, and profoundly heterogeneous concept of holistic wellbeing^3^ can only take shape within an urban space that guarantees an high quality of living. The objective of age-friendly cities has been progressively affirmed, emphasizing urban habitats (re)thought and (re)designed to be accessible, functional, welcoming, and desirable for their older inhabitants as well.

The complexity and processual nature of the ongoing transformations make scientific and political-administrative reflection navigate a challenging balance between the need to recognize the specificities of this phase of life and the imperative to avoid slipping into ageist stereotypes and prejudices (Seggio, 2022). This difficult yet necessary balance is indispensable for active inclusion policies to concretely shape awareness of the differences and peculiarities of the needs and desires carried by this complex group of citizens. In light of these needs, urban space must be designed as differential and inclusive space.

Building on the perspective outlined in the international action plan in Madrid (2002), cities have become key factors in crafting a new narrative aimed at promoting not only wellbeing and economic inclusion but also participation and social inclusion in its broader sense (Walker and Foster, 2013). The right to participation, closely linked to social inclusion, is functional to the pursuit of goals and strategies for the active aging of the elderly population (Walker and Maltby, 2012; Ekerdt, 2002; Bruggencate et al., 2018; Carrera, 2024), as well as the right to full social and political citizenship (Carrera, 2020).

Within broader urban policies aimed at this objective, as part of a reflection on the wellbeing of elderly individuals, a fundamental role is played by policies to counteract relational poverty. The elderly, in fact, are more exposed to the risk of both real and perceived loneliness. The reasons are diverse, including the lengthening of life expectancy, not necessarily accompanied by enduring health quality, and different residential choices, leading families to often live far from their place of birth. In the absence of adequate public policies, the quality of life for elderly individuals depends largely on their personal socioeconomic and cultural resources. To mitigate this effect, the role that public welfare can play in achieving the goal of ensuring widespread wellbeing, understood in a holistic perspective as a complex outcome of the combination of material and immaterial, individual and social elements, is crucial. Within this process, the quality and intensity of relationships have progressively assumed centrality, and when low, they are considered a genuine form of poverty. These new perceptions of wellbeing are influenced by the change in social representations and self-representations of the elderly, even if involve only a part of the individuals and their families.^4^

Relational poverty represents one of the most dangerous factors of social vulnerability since, on the one hand, it can significantly impact the quality of life for individuals, and, on the other hand, it is less emphasized compared to more classic economic poverty, for which measures and incentives are more easily activated (Esping-Andersen, 2000). Consequently, it risks not being perceived as a true priority in the representations of public administrators or in those of elderly individuals, ending up being conceived of as an entirely individual problem to be solved, if possible, on the same plane. With reference to one of the clear contradictions noted by the German sociologist Ulrich Beck (1999), often, individual solutions are sought for systemic problems, with the result of making the political, as well as collective, nature of the issue of loneliness among the elderly less evident. This ends up shifting the problem to a personal level, weakening its recognition as a social issue. This condition, which risks, as in the past, continuing to be considered natural for those experiencing the third phase of life, seems to need to be approached with patience and even resignation.

It is the reevaluation of the third and its social representation that is progressively transforming relational poverty, even that of the elderly, into a social problem. This means that public institutions, within a broader project of creating the conditions for widespread wellbeing, are called upon to specifically and urgently address the contrast to this “new poverty.” The aim is to prevent the quality of life for elderly individuals from depending solely on personal conditions and individual resources. In this perspective, urban space, both as public and private space (Carrera, 2020, 2023), can represent a key element as a strategic lever to counteract the risks of isolation and their repercussions on wellbeing and quality of life. With reference to public space, drawing on a neo-conflitaliast and neo-materialist perspective also embraced by Harvey (2016) concerning the right to the city, the quality and material characteristics of urban space can significantly influence its usage practices and the opportunities available to individuals, especially those with limited mobility, who are more reliant on the characteristics of their immediate surroundings.

## 3 The functions of spaces

Nigel Thrift observes that built space is “actively passive” (Thrift, 2016). Once constructed, in this perspective, even cities continue to influence the possibilities of social relations and different levels of power and inequality (Greenfield et al., 2019). The city's shape impacts the structural conditions of the everyday experiences^5^ of the third and fourth ages to guarantee the full right to active aging and a high level of quality of life, even within urban spaces. To achieve these objectives, cities should increasingly adopt public policies focused on counteracting relational poverty (Zajczyk, 2018). This involves moving beyond considering “societal resources invested in the betterment of aging as a drain on the economy more than a productive investment having both tangible and intangible benefits” (D'Souza, 1993, p. 342). Therefore, it is a matter of going beyond the ethical, social, and political dimensions of the need to guarantee full citizenship and a high level of quality of life for elderly individuals, toward the awareness that pursuing these goals also has significant economic implications, including savings in resources allocated to healthcare and welfare. The fullness of daily life also positively influences health and delays the deterioration of autonomy, as well as the subsequent decisions to admit individuals to nursing homes and long-term care facilities. Therefore, working on and designing the quality of urban space means investing in the quality of life for individuals, but also in the long-term savings of public resources. The organization of public space and the suitability and availability of mobility infrastructures across the city are crucial in defining the quality of life, social relations, and sense of community for different types of citizens, especially those who are socially fragile and vulnerable, such as older adults (Bonoli, 2004; Emerijck, 2002; Esping-Andersen, 2000; Pavolini, 2001, 2004; Zajczyk, 2018).

In this line of thought, and within the analysis of the centrality of public space in creating conditions and opportunities for relationships, the concept of the Third Space becomes particularly relevant. Edward Soja, building on Lefebvre (1967; 1975/1991)^6^ reflections on the social production of space and recognizing its primacy in the consideration of the “Thirdspace,” conceptualizes it as a set of new spaces that are somewhat liminal, interstitial, within which conditions for encounter and mutual recognition can be created. Thirdspace is an analytical concept that opens up to a condition of multifunctionality and the ability to be places of gathering and welcome. These could be workplaces, schools, universities, youth centers, sports clubs, public libraries, museums, or other places repurposed for this specific goal, such as community social centers or neighborhood houses. They can be thought of and experienced as places where conditions for shared activities and even collaborative planning can take place (Amore and Hall, 2016). “A space of radical openness, a vast territory of infinite possibilities and perils. This space was not located simply ‘in between' his bi-polar worlds of centers and peripheries, or in some additive combination of them. It lay ‘beyond' in a (third) world that could be entered and explored through metaphilosophy” (Soja, 1996, p. 33).

As observed, public space, and particularly third spaces, understood as self-spaces of prolonged and repeated encounters, have the potential to host fundamental social and cultural infrastructures. These infrastructures represent a decisive element in ensuring access to opportunities that reinforce the level of wellbeing, especially for those individuals, like the older, who are more connected, sometimes even bound, to the territory in which they reside. This has the consequence of improving the quality of life on both a physical and an intellectual and emotional level.^7^ These levels are deeply interrelated, as numerous studies have demonstrated, with the overall quality of wellbeing experienced by elderly individuals capable of even slowing down the aging process in terms of the decline in physical and cognitive faculties.

Recognizing the quality of public space as a focal factor in ensuring the right to active aging,^8^ policies are called upon to respond to two fundamental principles: social justice and territorial democracy (Moreno, 2020; Carrera, 2020; Slughter Brown, 2017; Secchi, 2013).^9^

When the presence of these and other types of accessible and well-structured spaces is widespread across a territory, it shapes the right to *aging in place* (Wiles et al., 2011; Morganti, 2022; Robison et al., 2012; Pani-Harreman et al., 2000). In this sense, elderly individuals do not need to move from their place of residence to access greater opportunities. In order to guarantee the ageing in place is essential the role of friendly networks and neighborhood support in the process of active aging. Therefore, it is necessary to preserve these networks to ensure the persistence of a fundamental factor for the quality of life and wellbeing of older individuals. This right is also particularly evident in the connection with one's own home. Losing that connection can result in a sense of displacement that influences the feeling of wellbeing and quality of life.

At the same time, it cannot be overlooked that, the home can become a trap when individuals lacking social networks find themselves living in isolation. But in the presence of high-quality social networks, it can become the place of identity roots from which to move in order to open up to relationships and new opportunities. The home, a symbol of stability and security but also of privacy and family, has been increasingly studied as a potential hub of family and friend relationships within a broader network of social relations.^10^

Alongside policies that requalify public space to make it more accessible and usable by older citizens, those supporting the redesign of private space and forms of living are also crucial. It is no longer possible to think of living as determined by the dichotomy between the public space and private one recognizing them as interconnected through a deep and complex relationship, between the objectivity of a functional system and the subjectivity of a nonfunctional and symbolic system (Boni and Poggi, 2011).

## 4 Cohousing and combating relational poverty

While avoiding reinforcing ageist prejudices (Moulaert; Attias-Donfut and Tessier, 2005), it is essential to recognize that older individuals are more exposed to the condition of loneliness, which can even become structural, with severe consequences for their overall wellbeing. In addition to public space, private space represents an important element in ensuring conditions for a quality living environment that shapes the everyday lives of older individuals. Private living space could and provide opportunities to prevent and counteract the conditions of loneliness and relational poverty.

In the perspective of the role that the quality of housing can play in ensuring the conditions for social relationships, it is important to reflect on the specific experience of cohousing. The focus of this new and different form of living involves private rooms linked with shared and more or less wide common spaces within private houses of counteracting relational poverty.

The complex nature of these objectives explains the need for coordinated interdisciplinary and multifactorial actions in which public welfare, social cooperatives, and housing associations work together to enable well-structured territorial networks to create a functional urban habitat.

Sometimes solutions can be offered to people with disadvantaged socioeconomic backgrounds, although it is crucial not to confuse cohousing with the phenomenon of social housing aimed at individuals in conditions of marked economic distress.

Cohousing originated in Northern Europe in the 1970s as an evolution of the Swedish “kollektivhus” from the mid-twentieth century, which featured common spaces and services to help working women balance work and family. In Denmark, in 1964, architect Jan Gødmand Høyer used the term “bofælleskaber”—living community—to describe a new idea of dwelling rich in satisfying neighborhood relations. After the Danish project Skråplanet in the 1970s, this new housing model began to gain traction, especially in Northern Europe (Denmark, Sweden, and the Netherlands), and from the 1980s onwards, in the United States. The different demographic structure and, above all, a profoundly different culture mean that cohousing started gaining traction in Italy only during the 2000s, and mostly in the Northern regions of Piedmont, Emilia-Romagna, Lombardy,^11^ and Tuscany.

Despite the increasing number of cohousing experiences, the numbers in Italy are still low, with about fourthy experiences, and they mostly arise from private initiatives, often not supported by public administrations, as is the case in other European countries where renting is also an option. One of the few exceptions is “Porto 15” in Bologna, a public cohousing offering rental spaces for a specific period to young startups and innovation hub operators.

The specificity of cohousing lies in scaling down the sense of community action, which materializes in social action and the strengthening of relational networks. The mechanism of reciprocity prevails in the experience of cohabitation and neighborhood communities, relying on the sentiment of “brotherhood,” according to Weber. The shift toward a “cooperative of inhabitants” not only serves as a marketing strategy but also effectively configures a different cultural elaboration of dwelling and its social declination.

Even the dramatic experience of the pandemic has sparked a new interest in this possibility, reducing isolation and ensuring sharing not only of spaces but also of time, services, and, above all, activities and opportunities for social interaction. However, it remains a niche experience, undertaken by few due to bureaucratic and practical difficulties and, more importantly, cultural resistance, despite a high level of interest in the different conditions of living offered by this specific form of social housing.^12^ These variables are crucial, as they heavily impact one of the key elements of cohousing, namely, the choice of cohabitation (Gresleri, 2017).

As stated on the main page of the Housinglab website,^13^

When housing units not only offer a basic solution (housing) but also integrate services within them that promote relationships between neighbors for the management of everyday life, they can be defined as collaborative housing (Rogel, 2013; Rogel and Corubolo, 2012). They are participatory and accessible homes, innovative and inclusive, combining, in private spaces dedicated to individual households, places and services for sharing, exchanging, and conscious consumption. Spaces and moments where people collaborate to overcome small everyday difficulties and create a more enjoyable and consistent urban life with their own value system. In all examples of this type of housing, collaboration among residents is an inseparable part of the housing model.

Although cohousing never entirely coincides with social housing, it offers economic advantages. Housing costs in projects like Homers are on average 10% lower than market prices. Regarding energy savings, which clearly depend on the use of shared spaces, the estimated average is around 15%, although the installation and maintenance of renewable energy sources—such as solar panels—shared among cohousers can significantly increase economic savings on expenses. The specificity of cohousing, however, is found in the advantages at the social and relationship level, countering the widespread dynamics of fragmentation and isolation reflected in situations of profound loneliness.

Cohousing can thus represent a fundamental strategy to combat relational poverty by creating new forms of sociality and *active neighborhood engagement*. Sharing common spaces and even their management can facilitate a sense of belonging to a real community. The collaborative living factor is crucial for achieving the goal of improving the overall quality of life. The principle is to enhance housing and proximity as an opportunity for solidarity and communal living based on the collaborative living model.

In Italy, a central obstacle to the potential diffusion of this still experimental form of living is the lack of a law that makes these residential models a legal entity in every respect and simplifies their feasibility.^14^ As some associations concerned with this issue have noted, there is a proposed law for the recognition of intentional communities currently stalled in Parliament.^15^

Despite the concentration of these social experiments mainly in Northern Italy, there is a growing diffusion of these innovative experiences in the Central and Southern regions, indicating an increasingly widespread interest in new forms of living and (re)generating relationship forms. It is interesting to note that some of these experiences target specific groups, such as the cohousing dedicated to autism in Rome called “Spazi Solari,” born from the needs of a group of parents of autistic adults and children and realized in collaboration with various organizations long involved in the field of intellectual disability, including Il Filo dalla Torre, Etica e Autismo, Dhyana, and Accademia Peac. Another project in Perinaldo (Imperia) is dedicated to people with severe disabilities close to being deprived of family support. The project envisions the creation of five family-type housing solutions and social cohousing, in addition to an existing accommodation for the support educator, along with the refurbishment of spaces for common activities. In Bari, supported by the Welfare Department of the Municipality, the “Housing Lab” project has also been launched to assist vulnerable individuals in finding housing.^16^

The interest aroused in Italy by this new form of living has translated into the activities of numerous associations that conduct mappings and monitor social living experiences. This is aimed at identifying and sharing best practices and intervention models, both more structural and more connected to daily experience.^17^ A more recent example in Puglia is the mapping and monitoring experience carried out by the national research AGE-It, coordinated at local level (Spoke 7) by Letizia Carrera. Among others, one goal is to identify and analyze institutional and spontaneous cohousing experiences.^18^ Among the explicit objectives is that of differentiating and mapping these experiences based on specific characteristics and variables, ultimately generating a catalog of best practices and, in the end, integrating these experiences into a systematic framework.

Cohousing, therefore, is not just a new housing model but a genuine new lifestyle that ensures an active existential dimension and represents a worthy possibility for maintaining adequate levels of autonomy and quality of life. This type of living, often experienced by young students, can also be extended to older people, allowing them to experience new patterns of encounter and relationships.

### 4.1 The potential of senior cohousing

Senior cohousing is a variation of the broader cohousing model, involving self-managed residential communities specifically created by and/or for seniors. It significantly differs from traditional models of nursing homes or assisted living facilities due to the active presence it requires of the involved individuals. It is a collaborative housing model designed for seniors, aiming to provide a housing solution that meets their specific needs while promoting an active, participatory, and socially integrated life. It is crucial to distinguish between spontaneous initiatives and assisted initiatives supported by institutions, socio-health services, or third-sector cooperatives and private social entities. In the latter case, cohousers, called to be fully active in their living arrangements, may be supported by institutional operators or managers in various phases of organizational and decision-making processes, especially when projects are financially supported by public administration, and managers are required to chart their progress and account for expenses.

Senior cohousing represents an innovative approach aimed at providing seniors with a healthy, active, and socially engaged living environment, promoting participation and solidarity. The advantages of this specific form of living include the following: (a) reducing the risk of social isolation within residences, especially for individuals with limited mobility and low levels of economic and relational resources; (b) serving as a significant alternative to choices of institutionalization or reliance on home caregivers (known in Italian as “badanti”), who often lack the necessary skills and end up exacerbating the condition of closure and isolation among elderly individuals; (c) overcoming situations of loneliness, even abandonment, and socioeconomic hardship through the reception of this model of shared homes; (d) creating or reinforcing friendly and relational networks; (e) involving older individuals in decision-making and practical processes; (f) promoting greater self-care in terms of physical and psychological health; and (g) playing an active role in supporting the social plan shared with Territorial Social Services (Carrera, 2022). These benefits can be discussed into four macro types: (1) promoting self-management of an active life; (2) creating a sense of community; (3) social and environmental sustainability; and (4) integration with the territory. The first macro type includes (1a) *Self-management and active participation*: cohousing community members actively participate in community management, often basing decision-making on a consensus model, encouraging the active involvement of all residents; (1b) *Participatory design*: future residents are often involved in the design and construction of structures; (1c) *Independence and active living*: senior cohousing aims to promote an active and independent lifestyle for seniors, with common spaces facilitating accessibility and providing opportunities for social and recreational activities; and (1d) *Open communication and shared management of issues*: open and transparent communication is fundamental within these communities, facilitating participatory management and the collaborative resolution of any issues. The second macro type offers (2a) *Strong sense of community*: cohabitation among people of the same age group creates a strong sense of community, countering the loneliness often experienced by seniors. For the third macro type, we find (3a) *Resource sharing*: a key principle of senior cohousing, including common spaces, facilities, and services; this model also encourages the reduction of individual costs and promotes a sense of community; (3b) *Social and environmental sustainability*: senior cohousing communities foster a culture of mutual support among residents, and in some cases, these communities may integrate environmentally sustainable practices into the design and eco-friendly management of structures; (3c) *Adaptability*: senior cohousing communities are designed to be adaptable to the changing needs of seniors over time, allowing for modifications to spaces to make daily life more manageable and ensuring accessibility. The benefits related to the fourth and last macro type encompass (4a) *Care and support*: although aimed at promoting independence, senior cohousing can integrate assistance or care services to ensure support when needed; all communities encourage mutual support among seniors, including practical help, emotional support, and sharing experiences, and some may integrate assistance or home care services, ensuring that seniors can receive the necessary support without leaving the community; and (4b) *Access to services and activities*: proximity to cultural, recreational, and medical services and activities can serve as an important criterion in choosing the location of senior cohousing communities.

Senior cohousing experiences in Italy, face challenges that can vary depending on specific projects and local dynamics. These challenges can significantly differ from project to project, and effective management of these challenges requires attention, resources, and continuous collaboration between community members and local stakeholders.

Considering, as before, this list as open, the main problematic issues can be grouped into three major macro types: (1) interpersonal challenges; (2) systemic-environmental challenges; and (3) open challenges. Concerning the first macro type, we can include (1a) *Conflict management*: living in a communal environment can lead to differing opinions or conflicts; effective conflict management is essential for maintaining harmony within the community. (1b) *Funding and economic sustainability*: maintaining the economic sustainability of communities can be challenging; initial funding and ongoing management of common finances can generate tension and cause conflict. (1c) *Participation and involvement*: ensuring active participation by all members can be complicated; some residents may not be interested or able to actively participate in all decisions related to daily management. (1d) *Demographic variations*: changes in the community's composition due to new arrivals and the death of residents can influence community dynamics and cohesion. (1e) *Differences in vision and goals*: seniors within the community may have differing visions regarding goals and how the community should operate, leading to conflicts and decision-making challenges. (1f) *Design and structures*: participatory design can lead to differences of opinion on the arrangement of common spaces or housing unit design, generating potential tensions among residents. (1g) *Financial resources*: sharing resources can lead to financial issues if some residents contribute more than others or if the management of shared finances is unclear and non-transparent.

For the second macro type, we can identify (2a) *Laws and regulations*: senior cohousing arrangements may face challenges with local regulations regarding land use and construction; adapting existing structures or obtaining approval for new projects can be an obstacle. (2b) *Social acceptance*: some cohousing projects may encounter resistance from the surrounding community or local authorities; social acceptance is important for the success and stability of these initiatives. (2c) *Access to care services*: not all cohousing communities have easy access to assistance or home care services; ensuring access to support services can be crucial for seniors in need. (2d) *Home care assistance*: while some communities integrate assistance services, there may be a need to address issues related to the adequacy and continuity of home care services. (2e) *Changes in health and autonomy*: the health needs of seniors can change over time, requiring adjustments to structures and services to ensure the community can continue to meet their needs. (2f) *Economic sustainability*: the long-term economic sustainability of the community may be a concern, especially if there are fluctuations in financial resources or if the structure is unable to attract new members.

And, finally, among the challenges, we can mention (a) *Commitment and active participation*: participation in the cohousing's daily routine can pose a challenge for seniors who may prefer a higher level of independence and/or may not be interested in or capable of actively contributing to community management. (b) *Social isolation*: despite efforts to promote an active social life, some seniors may experience social isolation if they do not participate in common activities or if the community is not well integrated into the broader context. (c) *Adaptability to needs*: the adaptability of structures and services to the changing needs of seniors can be a challenge, especially when health conditions change. (d) *Integration with the local community*: integrating cohousing communities with the broader social fabric can be a challenge; inclusion and interaction with the local community are important to avoid isolation. (e) *Environmental sustainability*: while some cohousing projects may adopt sustainable practices, ensuring long-term environmental sustainability may require continuous commitment and resources.

Among the main cohousing experiences in Italy involving seniors, intergenerational projects in the Quarto Oggiaro neighborhood in Milan, named “Cascina Arzilla,” involve both seniors and young people in a communal environment with the goal of promoting solidarity and resource sharing. Projects in Bologna involve seniors and families with children, aiming to create an environment where different generations can coexist and support each other. More specific are the senior cohousing projects initiated in Tuscany, aiming to create autonomous and participatory communities that allow seniors to age in a sustainable and socially active environment. Examples include “Vivi per un Sorriso” in Ferrara, aiming to create shared housing for seniors desiring a supportive and friendly environment, and the senior hotel community in Modena, transforming a former hotel into a community for seniors with shared services and active participation. Other experiences include the Algarve Residence in Lucca, offering assistance services and a supportive community for resident seniors, and initiatives in Bolzano promoting active and participatory lifestyles through senior cohousing.

Although the geographic distribution of these senior cohousing practices appears to suggest a kind of divided Italy, with a clear prevalence of initiatives in the Northern regions, there are also notable experiences in other parts of Italy. One such project is “CondiViviamo,” an ongoing experiment in the metropolitan area of Bari, representing the first cohousing project in Puglia specifically dedicated to individuals over 65.^19^ The project serves as a laboratory to activate further experiences in the Puglia region. The project aims to promote an active aging model through cohousing and self-help practices involving older people. The goal of this project is to propagate a new lifestyle that ensures better wellbeing and a higher quality of life and encourages the creation of social networks among the elderly.

The project's stated objectives include (a) combating loneliness in daily private space; (b) strengthening self-empowerment strategies through direct collaboration in apartment management and related shared living activities; (c) optimizing housing costs; (d) ensuring the maintenance of spatial references and friendship networks based on the distribution of project-involved houses in the metropolitan area of Bari; (e) facilitating access to socio-health services, including home services; and (f) providing almost daily support from cooperative operators responsible for the project. The project has also highlighted challenges that require attention in planning subsequent experiments, including issues related to living with unfamiliar individuals, uncertainty about establishing functional relationships, and limitations associated with the availability of apartments that allow for the extensive spatial distribution of residences and the possibility for the individuals involved to make living choices by leveraging existing friendship and territorial networks.

Among the more specific elements that have made the management of this specific project more complex and even critical, there are, the modalities through which individuals were assigned to the cohousing experience. In the case of the “CondiViviamo” project, it was Social Services that, because of the centrally funded nature of the project, sent severely fragile individuals, whose presence prevented subsequent entries of a different type. This obligation led to the accommodation of seniors with similar characteristics (former prisoners, residents of family homes, individuals from public dormitories, etc.), removing the element of choice that is fundamental to cohousing experiences. This situation ended up accentuating the normal difficulties of coexistence among individuals. Relational problems (a high level of conflict that required almost continuous mediation by the cooperative staff) and those related to the management of apartment care activities (including the need for two pest control interventions due to bed bug infestations) discouraged other interested individuals from participating in the cohousing experience, which limited the project's outcomes. The characteristics and habits of the individuals made it impossible for the cooperative managing the project to conduct the necessary compatibility assessments and organize suitable living arrangements.

## 5 Conclusions

The cohousing theme is undoubtedly central to addressing the widespread housing problem affecting a growing number of individuals, not only seniors, who face difficulties in accessing credit and encountering the resistance of landlords to renting apartments, now perceived as a risk. More importantly, cohousing is central to addressing the issue of relational poverty, which, beyond any ageist interpretation, has become one of the major psychological and physical risk factors for seniors, significantly affecting their wellbeing. The regulatory weaknesses observed in Italy, due to the gap between international regulations and their implementation in national legislation and the high regional autonomy within national legislation,^20^ contribute to making this housing experience still relatively uncommon and profoundly differentiated especially with reference to the older individuals (Ghisleni, 2017). This is especially concerning given persistent challenges hindering the spread of a housing formula that appears to be entirely consistent with the changed features and needs of new demographic scenarios and expectations regarding the quality of life and wellbeing of socially marginalized categories of people. Among the effectiveness challenges to the cohousing formula, it is important to note the need for a multi-actor network that must be coordinated to ensure continuity for initiatives that require support before becoming self-sustaining. The role of institutions is central in this regard. This element could be supportive in the face of a still limited culture of cohousing that struggles to attain independent visibility and attractiveness, where institutional economic support could be a highly impactful factor (Wise, 2004).

Recognizing the centrality of relational poverty within the broader theme of the right to a high quality of life shifts support for these types of projects within the framework of institutional responses to the diverse needs of individuals for the realization of an inclusive territorial welfare (The Care Collective, 2021; Carrera, 2020). For this reason, in some cases, cohousing projects should be “tailored” to meet the specific wants and needs of the cohousers themselves, who directly collaborate with designers and architects on their development in a participatory design logic.^21^

Finally, it is worth considering that housing is both a private and a political matter at one and the same time (Perini, 2020, p. 189).^22^ In this sense, cohousing, as a method of producing innovative forms of shared space, can represent a potential strategy for revitalizing small neighborhoods and achieving a widespread and high level of quality of life toward an age-friendly and inclusive city (Carrera, 2020, 2021).

## Footnotes

^1^The number of people over 80 is also growing, reaching 4,529,000, representing 7.7% of residents, while the number of centenarians has tripled since the beginning of the new millennium.

^2^According to a UNESCO report, by 2050, the population living in urban areas will reach 68% of the total.

^3^“It involves a wider notion of wellbeing, both experienced and perceived, and less as a circumstance linked to health in the strict sense (Caradec, 2001a,b; Bai, 2014; Carrera, 2020). (…) The recent Agenda 2030 of the United Nations has fully acknowledged this new political and social centrality of older people among its objectives, which include a focus on their right to an inclusive and accessible city and recognize urban spaces as physical structures that can deeply affect their daily life. It's a focus goal to make social and/or physical environments more conducive to older adults' health, wellbeing, and ability to age within their places and their communities (Greenfield et al., 2019)” (Carrera, 2023).

^4^Despite the ongoing transformative processes redefining old age, there are still many who adhere to traditional representations and continue to perceive old age as a condition to be lived and “endured” with few expectations and a lot of patience (Gilleard and Higgs, 2002).

^5^“As a structuring device, the built space organizes and defines practices, thus contributing to the reproduction of social relationships. Both the purely physical dimension of spatial organization and the symbolic one, therefore, contribute not only to ‘suggest' certain uses of space and specific social relationships, but also to give concrete form to the life that takes place there. (...) The physical environment is linked to the knowledge and the dominant powers in the society by a mutual connection” (Amendola, 2018, p. 39, 43).

^6^“Without ever using the specific term, Lefebvre was probably the first to discover, describe, and insightfully explore Thirdspace as a radically different way of looking at, interpreting, and acting to change the embracing spatiality of human life” (Soja, 1996, p. 29).

^7^The sector literature reports numerous studies that have confirmed this connection, which has now become well-known even outside the medical field.

^8^Furthermore, it is worth noting that a substantial body of literature within the critical gerontology research stream (Van Dyk et al., 2013; Seggio, 2022; Boudiny, 2013; Van Dyk, 2014; Calasanti, 2021, 2008) seeks to problematize the same categories of successful and productive active aging, used both in classical gerontological and political contexts in response to the progressive and differentiated aging of the population.

^9^The first refers to the necessity for services and opportunities offered to citizens to be diversified and accessible so that they can be enjoyed regardless of individuals' economic, cultural, and social resources. The presence of “fee-based” opportunities, in fact, excludes all those who cannot afford them, and the same applies to proposals that involve cultural competencies not necessarily possessed by everyone. The second principle, that of territorial democracy, instead refers to the need for such offerings and opportunities to be widespread in urban spaces, thereby overcoming the limitation of the divide between resource-rich and resource-deprived areas and thus limiting the impact of the real symbolic peripherality of places (HERE).

^10^With the transformation of the housing (and urban) form, we can grasp the meanings of dwelling and thereby the social transformations as they have historically emerged. In this sense, the home is an important “archaeological site” for understanding how society changes: “The home is a prime unexcavated site for an archaeology of sociability” (Putnam, 1994, p. 144).

^11^In 2006, Cohousing.it started to develop in Milan, a company of professionals acting as an intermediary in the market, and it developed the first Italian example, the Urban Village Bovisa.

^12^In 2014, the “HousingLab” Association (https://www.programmaintegra.it) organized “Experimentdays – Collaborative Living Fair” in Milan, the first fair of collaborative living in Italy, and an important test to assess the interest in bringing innovation into a sector like real estate, where changes are very slow, despite the urgency of the housing theme and related sectors: care for people, active aging, energy efficiency, and more. The success of exhibitors and visitors during the 2-day fair confirmed this hypothesis and provided an opportunity to start building awareness around a collaborative living supply chain that not only includes designers and builders but also expands to involve residents, neighborhoods, and collaborative service providers.

^13^ https://www.housinglab.it

^14^Essential would be, for example, the possibility of accessing a bank loan without having to form a cooperative or paying the garbage collection tax without being considered a hotel.

^15^An initiative that started in the Netherlands in 2009 as “Intentional Communities Day” and later spread to other Northern European countries and now also in Italy. The goal is to promote and disseminate it to make it more known.

^16^As stated on the website of the Municipality of Bari, After a co-design phase, which saw the administration alongside Artes in the analysis of expressed needs, we moved on to disseminating information about the project's objectives to the Municipalities, local services, third-sector organizations, community houses, and SPRAR, which represent the main antennas for intercepting housing difficulties. Actions began concretely last May, and to date, there are about twenty individuals, both Italians and foreigners, identified by social workers and interested in being included in the project. For each case, factors such as the income received, the number of people in the household, willingness to cohabit, distance from the workplace, the possibility of having pets, or sharing spaces for smokers are taken into account.

^17^Among these, for example, there is HousingLab (https://www.italiachecambia.org), a nonprofit association founded with the aim of spreading good examples, sharing expertise, and experimenting with social and collaborative living. In particular, HousingLab deals with new housing models for new family models; collaborative services for affordable living; urban communities; public and private spaces; regeneration of existing housing contexts; social, environmental, and economic sustainability of homes; and social, environmental, and economic sustainability of relationships. To achieve these objectives, the Association aims to carry out the following nonprofit activities: (a) Promote and organize cultural activities such as conferences, fairs, exhibitions, seminars, competitions, events, and visits on the theme of social and collaborative living. (b) Promote, organize, and provide training activities, including theoretical and practical courses, workshops, also with an educational focus in schools of all levels and universities, even for nonmembers. (c) Create a network of national and international stakeholders for sharing and exchanging ideas and experiences on relevant issues. Through constructive dialogue, contribute to the development and dissemination of best practices. (d) Publish and disseminate articles, books, publications, and videos in paper or digital format on the theme of social and collaborative living.

^18^Research activities are carried out within the work program of the Urban Studies Laboratory Urbalab.

^19^The project “CondiViviamo” is managed by the social cooperative “Occupazione e Solidarietà” of Bari, in collaboration with the University of Bari Aldo Moro and financed by the Department of Welfare of the Municipality of Bari.

^20^Since the 1980s, the European Community has formulated a series of guidelines, suggestions, and best practices to address the issue of housing for the elderly. However, it is with the “package” of ministerial decrees (DM 2521-2524/2001) defined as the “Experimental Program of Residential Construction: Rental Housing for the Elderly in 2000” that Italy essentially introduced what later became the cohousing model.

^21^The reference to Homers' projects is significant.

^22^Perini (2020, p. 187) writes that it's about living and sharing rather than simply living together. The distinctions are not merely terminological and formal; instead, they express a different vision and perspective in the way living spaces are designed and used (Perini, 2020, p. 190).
